# MicroRNA Involvement in Osteosarcoma

**DOI:** 10.1155/2012/359739

**Published:** 2012-04-03

**Authors:** Eisuke Kobayashi, Francis J. Hornicek, Zhenfeng Duan

**Affiliations:** ^1^Department of Orthopaedic Surgery, Massachusetts General Hospital, Boston, MA 02114, USA; ^2^Sarcoma Biology Laboratory, Center for Sarcoma and Connective Tissue Oncology, Massachusetts General Hospital, Boston, MA 02114, USA

## Abstract

Osteosarcoma (OS) is the most common primary malignant bone tumor, usually arising in the long bones of adolescents and young adults. While our knowledge of the molecular pathogenesis of OS has increased in recent years, we are still far from a comprehensive understanding of the molecular mechanisms of the disease, such as its tumorigenesis, specific mediators of disease progression, occurrence of chemoresistance, and development of metastasis. After the recent discovery of microRNAs (miRNAs), their critical roles in molecular biological processes have been of great interest in the cancer research field, including research on sarcomas. MiRNAs are highly conserved noncoding RNAs which play important roles as oncogenic or suppressive genes to simultaneously regulate multiple targets. Recent genome-wide screening using miRNA expression profiles has identified specific miRNA expression patterns that are associated with the biological and clinical properties of cancers. Additionally, miRNAs and their target genes or proteins can be potential novel biomarkers or therapeutic targets for cancer. However, there are several challenges that must be addressed in order to translate miRNA-based therapeutics to the clinical setting. In this review, we summarize the current understanding of the roles that miRNAs play in OS, and highlight their potential as biomarkers or therapeutic targets.

## 1. Introduction

 Osteosarcoma (OS) is the most common primary malignant bone tumor with an incidence of 4 to 5 cases per million (approximately 900 newly diagnosed cases per year in the United States), mainly arising from the metaphysis of the long bones of adolescents and young adults [1]. The 5-year survival rate of the patients with OS has significantly improved over the past decades to approximately 60–70% since the introduction of combinatorial chemotherapy [2]. However, a significant proportion of OS patients still respond poorly to chemotherapy, and they have a risk of local relapse or distant metastasis even after curative resection of the primary tumor and intensive chemotherapy. Recently, novel molecular targeted drugs have emerged, but they have not been well established for the treatment of OS [1], in addition, the fundamental molecular mechanisms underlying the histological heterogeneity, drug resistance, and development of metastasis in OS remain obscure. Hence, it is essential to develop novel alternative strategies for the diagnosis, prediction of the prognosis, and treatment of patients with OS. 

**Figure 1:**
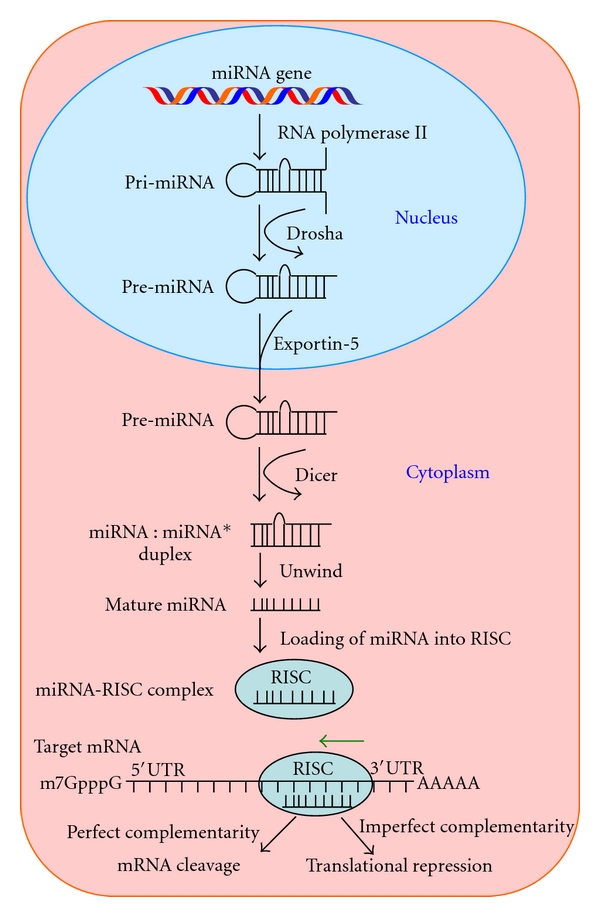
The biogenesis of microRNA in a cell. After the transcription of the miRNA gene in the nucleus, the primary transcript (pri-miRNA) is cleaved into a precursor molecule (pre-miRNA) with an imperfect stem-loop structure by Drosha. The pre-miRNA is exported from the nucleus into the cytoplasm by exportin-5. In the cytoplasm, the pre-miRNA is cleaved by Dicer into a dsRNA duplex (miRNA: miRNA*), which contains both the single-stranded mature miRNA and its complementary strand (miRNA*). The miRNA strand is then incorporated into the RNA-induced silencing complex (RISC) and targets the complementary mRNA sequences via translational repression or mRNA cleavage.

 MicroRNAs (miRNAs) are noncoding small RNAs, usually 18–25 nucleotides in length, which repress translation and cleave mRNA by base-pairing to the 3′ untranslated region of the target genes (Figure 1). They have the potential to regulate various critical biological processes, including the differentiation, progression, apoptosis, and proliferation of tumor cells [3]. Since the discovery of the first miRNA, *lin-4*, in *C. elegans *in 1993, it has been estimated that as many as 1000 miRNA exist in the human genome [4, 5]. More than 30% of the human genome is regulated by miRNAs simultaneously targeting multiple genes [6]. The recent differences in the miRNA expression profiles detected between cancer cells and their normal counterparts have revealed that miRNAs are involved in the pathogenesis of cancer [7]. In addition, miRNAs may play multiple roles as tumor suppressors, oncogenes, or both in some cases [8]. These biological properties of miRNAs may make them useful as diagnostic and prognostic tools in various cancers, including OS. In this review, we summarize the current knowledge regarding the involvement of miRNAs in OS (Table 1), with a focus on their potential application as therapeutic targets.

## 2. MicroRNA Expression Profiles

### 2.1. The MicroRNA Expression Profile of Sarcomas

miRNAs are endogenous RNAs that are highly conserved in the genomes of most species and can influence various biological processes, including the development and differentiation of tumors. To determine the expression pattern of miRNAs, a miRNA microarray approach has been developed. It has been reported that the miRNA expression profiles are able to classify human cancers successfully, whereas the messenger RNA (mRNA) expression profiles are inaccurate when applied to the same samples [9]. This result indicates that the miRNA expression profiles may be more closely linked to the differentiation of the tumors. Several studies have already demonstrated that there are unique and differential miRNA expression patterns for several cancers, which are promising for their diagnoses [7]. To identify novel miRNA-based biomarkers, the significance of miRNA expression profiles has been extensively studied in a diverse group of human sarcomas. For instance, Taulli et al. reported that miR-1 and miR-206 promoted myogenic differentiation to regulate skeletal muscle development, and blocked rhabdomyosarcoma tumor growth in mice xenografts [10]. The reexpression of these miRNAs in rhabdomyosarcoma cells induced myogenic differentiation and inhibited tumor growth. Recently, we also have identified miR-1 and miR-206, which are both expressed at a significantly lower level or are absent in chordoma cells compared to normal cells [11]. Reintroduction of miR-1 inhibited the growth of chordoma cells, with suppression of MET expression. MET is part of a receptor tyrosine kinase family of oncogenes overexpressed in many human cancers, including sarcomas, particularly in chordoma (94.4%), chondrosarcoma (54.2%), and osteosarcoma (23.3%) [12]. Importantly, a recent study suggested that the Met oncoprotein plays a major role in the metastatic process in chordoma [13]. Subramanian et al. obtained the miRNA expression profiles of 27 soft tissue sarcoma samples from 5 histological subtypes (synovial sarcoma, rhabdomyosarcoma, leiomyosarcoma, gastrointestinal stromal tumor (GIST), and liposarcoma) and 7 normal tissue samples [14]. In these expression profiles, different histological subtypes of sarcoma had distinct miRNA expression signatures, reflecting the apparent lineage and differentiation status of the tumors.

 With regard to OS, Sarver et al. have generated miRNA expression profiles for over 300 sarcoma tissue samples representing 22 different sarcoma subtypes (including 15 OS samples) and developed a sarcoma miRNA expression database (S-MED) [15]. Interestingly, an unsupervised clustering analysis of the miRNA expression profiles showed that OS formed a single cluster that was distinct from other sarcomas, such as synovial sarcoma, fibrosarcoma, GIST, and malignant fibrous histiocytoma (MFH) [15].

### 2.2. Dysregulation of miRNA in Osteosarcoma

 OS has histological heterogeneity and variability, having not only osteoblastic regions, but also chondroblastic and fibroblastic regions. The presence of a small area of tumor osteoid enables a pathologist to make the histological diagnosis of OS even when regions of fibrous tissue or cartilage are present [16]. Since a broad range of genetic and epigenetic alterations can be associated with the osteoblast differentiation pathway, osteoblasts have been considered as the origin of OS [17]. However, it has recently been demonstrated that the cells of origin of OS may be a multipotent stem cell (mesenchymal stem cells). OS is considered to be a differentiation disease caused by genetic changes that interrupt osteoblast differentiation from mesenchymal stem cells [17, 18]. In the miRNA expression profiles of OS, the formation of the single distinct cluster for OS indicates that miRNAs can regulate specific tissue-lineages during the differentiation of OS [14, 15]. In other cancers, recent studies also suggested that miRNAs may be involved in the development of tumors by critically regulating cancer stem cells, but their involvement in OS is still unclear [19].

 Several studies have investigated the role of microRNAs in OS using miRNA expression profiles. Maire et al. performed miRNA expression profiling of seven OS samples and suggested that the miRNAs provide a novel post-transcriptional mechanism for regulating the expression of specific pathways and genes related to OS [20]. Recently, Lulla et al. found twenty-two differentially expressed miRNAs in OS, and miR-135b, miR-150, miR-542-5p, and miR-652 were all highly expressed in OS tumor samples compared to normal osteoblasts [21]. Interestingly, miR-135b, which has already been shown to play a functional role in normal osteoblastic differentiation, was significantly increased in OS [22]. Stabley et al. also found several differentially expressed miRNAs, including miR-654 and miR-370, in OS xenografts. Interestingly, the *IRS1* gene is a predicted target of miR-370, and the IRS1 protein interacts with IGF1R, which is usually highly expressed in OS [23].

 The differential expression patterns of miRNAs in OS may be a consequence of the malignant phenotype, and may not actually be driving the biology of the tumor. However, these comprehensive analyses using a relatively high-throughput screening of the miRNAs will provide insight into the molecular mechanisms of OS.

## 3. *TP53*-Related miRNAs in Osteosarcoma

*TP53* is a well-known tumor suppressor gene involved in OS [24]. DNA damage induces the phosphorylation of p53, allowing it to dissociate from Mdm2, which leads to p53-mediated tumor suppression via cell cycle arrest or apoptosis. The *TP53* gene is mutated in more than 20% of OS, and its mutations have been demonstrated to be involved in the tumorigenesis of OS [25]. In addition, Li-Fraumeni syndrome, which is characterized by an autosomal dominant mutation of *TP53,* leads to the development of multiple malignancies, including OS [26].

 Recently, several miRNAs have been identified as direct targets of p53 [27]. Among them, the highly conserved miR-34 family (miR-34a, 34b and 34c) has been an important component of the p53 tumor suppressor pathway, and the expression of these miRNAs was induced by p53 in response to DNA damage or oncogenic stress in multiple cancers [28, 29]. Although the current knowledge about the involvement of p53-related miRNAs in OS is limited, He et al. reported that the miR-34 family induced G1 arrest and apoptosis via their targets, CDK6, E2F3, Cyclin E2, and BCL2, in a p53-dependent manner in OS cells [30]. According to an examination of the expression of genetic and epigenetic alterations of miR-34 in 117 primary OS samples, the expression of miR-34 was decreased in OS, and miR-34 inhibited the p53-mediated cell cycle arrest and apoptosis in OS cells [30]. Additionally, p53 also induced the upregulation of miR-192, miR-194, and miR-215 in U2OS cells carrying wild-type p53 [31]. MiR-192 and miR-215 induce the expression of p21, and U2OS cells transfected with an expression vector for miR-192 formed significantly fewer colonies than those transfected with that for a control miR or miR-34a [31]. The loss of miR-31 was associated with defects in the p53 pathway, and overexpression of miR-31 significantly inhibited the proliferation of OS cell lines [32].

 A recent study focused on the role of miR-31 in regulating the development of metastatic disease. Valastyan et al. demonstrated that miR-31 was able to inhibit multiple steps in the metastatic development of breast cancer [33]. The silencing of the mRNA targets of miR-31, integrin-*α*5 (ITGA5), radixin (RDX), and RhoA, reduced local invasion and motility *in vitro* and decreased the development of metastases in a xenograft mouse model of breast cancer [33]. These results suggest that *in vivo* delivery of miR-31 may have potential for the prevention of disease progression or the development of pulmonary metastasis in OS patients.

## 4. MiRNAs as Potential Biomarkers and Therapeutic Targets in Osteosarcoma

### 4.1. The Involvement of miRNAs in the Cell Proliferation and Metastasis of OS

 OS is not only locally aggressive but is also a systemic disease. Patients with OS can have a relapse of their tumor or develop distant metastases even after systemic chemotherapy and aggressive surgery. Although lung metastases are the main cause of death in patients with OS, the mechanisms are still largely unclear [34]. Consequently, novel therapeutic targets that can hinder the tumor progression or the development of metastases have been investigated, as have prognostic biomarkers [35]. Since miRNAs have been implicated in the regulation of several important biological processes, some groups have focused on miRNAs as an innovative form of therapy for treating OS.

 It has been reported that miR-21 is aberrantly overexpressed in various cancers and is involved in the pathogenesis of cancers [36]. MiR-21 also induces cell proliferation, migration, and invasion by inhibiting the expression of tumor suppressor proteins in several cancers, such as breast, hepatocellular, and colorectal cancer [37–39]. The inhibition of miR-21 significantly reduced the lung metastasis of breast cancer cells *in vivo *[40]. Ziyan et al. demonstrated that miR-21 was also significantly overexpressed in OS, and the suppression of miR-21 decreased the invasion and migration in MG-63 OS cell lines [41]. RECK (reversion-inducing cysteine-rich protein with kazal motifs) was found to be a direct target that was negatively regulated by miR-21 in an OS cell line and human OS samples [41], and it suppressed the invasion of OS cells by decreasing the activity of matrix metalloproteinases (MMPs) [42].

 In our study on miRNAs and OS, we have examined the miRNA expression profiles between three OS cell lines and three osteoblast cell lines [43]. We found that miR-199a-3p, miR-127-3p, and miR-376 were significantly decreased in the OS cell lines as compared to osteoblasts, while the expression of miR-151-3p and miR-191 was increased. Among these miRNAs, overexpression of miR-199a-3p in OS cell lines was associated with a significant decrease in cell growth with G1 arrest. Furthermore, miR-199a-3p suppressed the expression of oncogenic and antiapoptotic proteins, MET, mTOR, STAT3, MCL-1, and BCL-X. This suggests that miR-199a-3p plays a significant functional role in regulating the proliferation of OS cells. In support of our findings, the expression of miR-199a-3p was significantly reduced in several cancers, and restoring the level of miR-199a-3p induced growth suppression via regulation of mTOR and MET [44, 45]. Taken together, these findings indicate that miR-199a-3p can prove to be a promising candidate for gene therapy, and that attenuated expression of this miRNA is not specific to a single tumor type.

 With respect to the development of pulmonary metastasis in OS, Osaki et al. compared the miRNA expression profiles between HOS and 143B OS cells (HOS cells transformed via v-Ki-ras, resulting in a high rate of metastasis) [46]. The expression of miR-143 was significantly decreased in 143B cells compared to HOS cells. Transfection of miR-143 by systemic injection of miR-143/atelocollagen complexes into 143B cells led to decreased invasiveness and suppression of lung metastases *in vivo*. These data are consistent with that from a previous report which demonstrated that the restoration of miR-143 reduced cell viability and induced apoptosis in OS cell lines via an antiapoptotic molecule, BCL-2 [47]. Reduced expression of miR-143 has also been reported in several cancers, such as colorectal, prostate, ovarian, gastric, and cervical cancer [48–52]. Therefore, miR-143 has been considered to be a tumor-suppressor miRNA. Chen et al. demonstrated miR-143 significantly suppressed colorectal cancer cell growth by inhibiting KRAS transformation [53]. Akao et al. identified that *EPK5* mRNA was one of the target genes of miR-143 in B-cell lymphoma cell line [54]. In addition, MMP-13 was identified as one of the miR-143 target proteins in OS by immunoprecipitation [46]. The expression of MMP-13 in an immunohistochemical examination in the tissue samples of OS patients with lung metastasis was relatively low compared to those without metastasis [46].

### 4.2. MiRNA Expression and Chemotherapeutic Response in OS

 Advances in chemotherapy have resulted in the most significant improvement in the outcomes for patients with OS. Without chemotherapy, the survival rate of patients with localized OS is less than 20% at 5 years, but systemic combined chemotherapy (doxorubicin (DOX), cisplatin (CDDP), methotrexate (MTX), or ifosfamide (IFO)) has enabled the 5-year survival rates to improve to approximately 60–70% [2]. Although the response to chemotherapy is one of the most important prognostic factors, more than 40% of all OS patients still respond poorly to chemotherapy [2]. Only a few therapeutic options have been established for these poor responders [1, 2, 55]. Moreover, no biomarker has yet been identified that discriminates between good and poor responders before the introduction of chemotherapy.

 MiR-140 is the first reported miRNA candidate associated with drug sensitivity in OS tumor xenografts treated with the chemotherapeutic agents DOX, CDDP, and IFO [56]. Song et al. revealed that miR-140 showed consistently high expression levels across all three xenograft models treated with different drugs. The overexpression of miR-140 caused chemoresistance to MTX and 5-Fluorouracil (5-FU) and suppressed cell proliferation, inducing G1 and G2 arrest in both U2OS and MG-63 OS cells, thus indicating that slowly proliferating or quiescent cells were more resistant to DNA damaging agents. In addition, miR-140 could negatively regulate histone deacetylase 4 (HDAC4) which interacted with p21 expression, resulting in 5-FU resistance.

 The same group reported another miRNA candidate that plays a significant role in the mechanism of chemoresistance. MiR-215 decreased the cell proliferation and induced G2 arrest and also increased the chemoresistance to MTX in U2OS cells and HCT116 (wt-p53) colon cancer cells [57]. Denticleless protein homolog (DTL) was identified as one of the critical targeted proteins of miR-215 using a bioinformatics approach. The reintroduction of miR-215 suppressed the expression of the DTL protein. The suppression of DTL by a DTL-specific siRNA reduced cell proliferation by inducing G2 arrest, causing a poor response to MTX [57]. Boni et al. reported that miR-192 and miR-215 influenced the sensitivity of colorectal cancer to 5-FU [58]. These miRNAs induced cell cycle arrest with the accumulation of p53 and its target genes *p21* and *p27* [58].

 Recently, Gougelet et al. examined miRNA expression profiles to determine the relevance of miRNA expression on the chemoresistance in 27 OS paraffin-embedded samples, cell lines and samples from a rat OS model [59]. According to the supervised hierarchical clustering, five candidate miRNAs (miR-92a, miR-99b, miR-132, miR-193a-5p, and miR-422a) showed a statistically significant ability to discriminate good responders to IFO from poor responders. The targets of these miRNAs detected by the *in silico* approach are involved in cell cycle regulation, invasion, and bone resorption through MAP kinase, TGFß and Wnt pathways.

 Such discoveries of new miRNAs to predict a response to chemotherapeutic agents in OS can potentially be used to stratify patients so that they can be treated with different preoperative chemotherapy regimens in the future.

## 5. MiRNA Targeting Therapy and Delivery

The miRNA-based therapeutic approaches for OS may involve two main strategies. One is to block the expression of oncogenic miRNAs using antisense oligonucleotides (anti-miR). Antisense oligonucleotides are single-stranded molecules that form direct bonds by complementary pairing and work as competitive inhibitors of miRNAs. The other strategy is to restore the expression of tumor suppressor miRNAs by introducing miRNA mimics. MiRNA mimics are synthetic oligonucleotides that are identical to the selected miRNA. Recently, other novel approaches have been developed to increase the binding affinity and stability of these oligonucleotides. Locked nucleic acids (LNAs) are a class of nucleic acid analogues. LNAs have a methylene bridge that locks the ribosome conformation and displays unprecedented binding affinity towards complementary single-stranded RNA [60]. MiRNA sponges contain multiple complementary 3′ UTR mRNA sites to a miRNA of interest and competitively bind to that miRNA [61, 62]. The advantage of miRNA sponges is that they are capable of targeting and inhibiting a family of miRNAs, which is in contrast to single miRNA targeting with antisense oligonucleotides. For instance, the inhibition of tumor suppressor miR-31 using miRNA sponges resulted in the induction of lung metastases by nonaggressive breast cancer cells [33]. In contrast to miR sponges, miRNA-masking antisense oligonucleotides (miR-mask) consist of single-stranded 2′-*O*-methyl-modified antisense oligonucleotides. MiR-masks effectively depress their target mRNAs as they are fully complementary to the predicted miRNA binding sites in the 3′ UTR of the target mRNA [63].

 Currently, the main route for delivery of miRNAs *in vivo* is systemic delivery via the intravenous or intraperitoneaal route [8]. However, the development of more efficient delivery of miRNAs to the target cells is essential for clinical applications, because such oligonucleotides are extremely hydrophilic, sensitive to RNAse degradation, and comparatively large, decreasing their ability to penetrate the target cells [64]. Viral and nonviral strategies have been developed to overcome these difficulties. The viral strategies have been widely used for efficient delivery of genes. Although lentiviruses cannot target specific cells, and residual viral components can be immunogenic or recombinogenic [65], effective delivery of miRNAs via a lentivirus has also been observed [66]. Nonviral strategies generally involve the use of oligonucleotides with chemical modifications, liposomes, polymers, and nanoparticles. Takeshita et al. established atelocollagen-mediated delivery for the safe and efficient systemic injection of oligonucleotides and demonstrated the suppression of OS lung metastasis in mice injected by miR-143 with atelocollagen [46, 67]. Nanoparticles also hold promise for the delivery of miRNA more efficiently to the target cells because of their unique properties, which are characterized by improved stability, size, and biocompatibility, as well as their self-assembly [68]. Recently, miR-34a delivered by the actively targeted nanoparticles significantly downregulated the survivin expression in a murine model of metastatic melanoma [69]. Despite these new discoveries, there are still several obstacles to overcome prior to the introduction of clinical testing of miRNA-based treatments.

## 6. Conclusions

 To date, several miRNAs involved in OS have been described. While specific miRNAs have been shown to be overexpressed in OS, several miRNAs are also downregulated, as described above. These results suggest that miRNA-based therapeutic strategies to restore or inhibit the expression of miRNAs can serve as a novel therapeutic option for OS.

**Figure 2:**
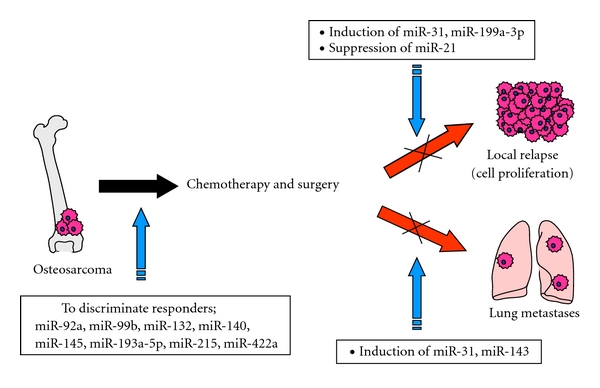
Potential candidate miRNAs to be used as biomarkers and therapeutic targets for OS.

 Altogether, the various studies indicate that miRNAs have the potential to be promising diagnostic biomarkers, as well as therapeutic targets, in OS due to their tissue specificities and critical roles in various biological processes (Figure 2). Moreover, miRNAs are detectable even in the sera of patients, leading to additional potential clinical applications. There has already been significant progress in the basic knowledge of sarcoma, which has unveiled the basis of many multiple biological processes. However, the research on the correlations of miRNAs with OS has just begun. Although miRNAs will undoubtedly contribute to the advancement of future clinical therapeutic applications for OS, further investigations and the establishment of ideal *in vivo *delivery systems will be essential.

## Figures and Tables

**Table 1:** List of microRNAs involved in regulation of OS.

MicroRNAs	Functions in OS	Expression level in OS samples	Analyzed OS samples	MicroRNA targets in OS	References
miR-34 family (miR-34a, 34b and 34c)	P53-related G1 arrest and apoptosis	Decreased	Cell lines and 107 tumor samples	CDK6, E2F3, Cyclin E2, BCL2	[30]
miR-31	P53-related cell proliferation	N/D	Cell lines	E2F2	[32]
miR-192, miR-215	P53-related cell cycle arrest	N/D	Cell lines	CDKN1A/p21	[31]
miR140	Chemoresistance to MTX and 5-FU	Increased in OS with chemoresistance	Mouse xenografts	HDAC4	[56]
miR-215	Chemoresistance to MTX	N/D	Cell line	DTL	[57]
miR-92a, miR-99b, miR-193a-5p, miR-422a	Discriminate good responders from poor ones	Increased	Cell lines and 27 paraffin-embedded samples	N/D	[59]
miR-132		Decreased			
miR-21	Cell invasion and migration	Increased	Cell lines and 8 tumor samples	RECK	[41]
miR-199a-3p	Cell proliferation and migration	Decreased	Cell lines and 12 tumor samples	MET, mTOR, STAT3, MCL-1, BCL-X	[43]
miR-143	Pulmonary metastasis	Decreased in OS with metastasis	Cell lines and 22 tumor samples	MMP-13	[46]

Abbreviation: N/D, no data; MTX, methotrexate; 5-FU, 5-Fluorouracil.
